# Vertebrate Reservoirs of Arboviruses: Myth, Synonym of Amplifier, or Reality?

**DOI:** 10.3390/v9070185

**Published:** 2017-07-13

**Authors:** Goro Kuno, John S. Mackenzie, Sandra Junglen, Zdeněk Hubálek, Alexander Plyusnin, Duane J. Gubler

**Affiliations:** 1 Formerly at the Division of Vector-Borne Infectious Diseases, Centers for Control and Prevention, Fort Collins, CO 80521, USA; 2 Faculty of Medical Sciences, Curtin University, GPO Box U1987, Perth, WA 6845, Australia; j.mackenzie@curtin.edu.au; 3 Division of Microbiology & Infectious Diseases, PathWest, Nedlands, WA 6009, Australia; 4 Institute of Virology, Charité-Universitätsmedizin Berlin, Helmut-Ruska-Haus, Chariteplatz 1, 10117 Berlin, Germany; sandra.junglen@charite.de; 5 Institute of Vertebrate Biology, Academy of Sciences of Czech Republic, 60365 Brno, Czech Republic; zhubalek@brno.cas.cz; 6 Department of Virology, University of Helsinki, Haartmaninkatu 3, University of Helsinki, 00014 Helsinki, Finland; Alexander.Plyusnin@helsinki.fi; 7 Program in Emerging Infectious Diseases, Duke-NUS Medical School, 8 College Rd., Singapore 169857, Singapore; duane.gubler@duke-nus.edu.sg

**Keywords:** insect-specific virus, arbovirus, transmission mechanism, vertebrate reservoir, origin of arbovirus, virus maintenance, zoonosis, host range

## Abstract

The rapid succession of the pandemic of arbovirus diseases, such as dengue, West Nile fever, chikungunya, and Zika fever, has intensified research on these and other arbovirus diseases worldwide. Investigating the unique mode of vector-borne transmission requires a clear understanding of the roles of vertebrates. One major obstacle to this understanding is the ambiguity of the arbovirus definition originally established by the World Health Organization. The paucity of pertinent information on arbovirus transmission at the time contributed to the notion that vertebrates played the role of reservoir in the arbovirus transmission cycle. Because this notion is a salient feature of the arbovirus definition, it is important to reexamine its validity. This review addresses controversial issues concerning vertebrate reservoirs and their role in arbovirus persistence in nature, examines the genesis of the problem from a historical perspective, discusses various unresolved issues from multiple points of view, assesses the present status of the notion in light of current knowledge, and provides options for a solution to resolve the issue.

## 1. Introduction

Despite the general impression that most of the fundamentals of arbovirology had been reasonably well established by the mid-1980s [1], a review shortly thereafter revealed significant gaps in understanding the roles of vertebrates in arbovirus transmission [2]. This was followed by the discoveries of other issues, such as nonviremic transmission, the extrinsic incubation period, the viremic threshold, and the reservoir status [3,4,5,6]. A further examination of the literature for this review raised even more problematic issues concerning the notion of the “vertebrate reservoir” in the arbovirus definition established by the World Health Organization (WHO) [1,7]. Recognizing and understanding these discrepancies is important, because arboviruses are defined by their unique arthropod-borne mode of transmission to vertebrate hosts, not taxonomically.

To help identify the genesis of the problems addressed in this review, we first briefly explore the historical events leading to the establishment of the basic principles of arbovirus transmission. Then we reexamine the data on which the notion of vertebrate reservoir was conceived, relate it to current knowledge, assess the impact on contemporary research to identify the issues that need reexamination, and, lastly, propose ideas to improve the arbovirus definition.

The WHO document was first published in 1967 and revised with minor modifications in 1985 [1,7]. In this review, however, we base the “arbovirus transmission concept” on only five subjects (viruses, vectors, vertebrates, the transmission mechanism, and the disease control). The term “notion” refers exclusively to the plausible assumption that vertebrates play a role as reservoirs in arbovirus persistence in nature, as defined in the WHO document. Similarly, “host” refers exclusively to a vertebrate species. The range of covered topics in this review is limited to selected issues surrounding the notion (persistent infection, the overwintering of the virus, the role of vertebrate hosts, the host range, zoonosis, and the origins of arboviruses) and excludes other factors traditionally considered to explain arbovirus maintenance such as re-introduction of virus. The vector–host relationships of arboviruses and closely-related viruses referenced in this review are listed in Table 1.

## 2. Genesis of the Notion of Vertebrate Reservoir

The transmission concept is built on four pillars: viruses, their vectors, vertebrate hosts, and the environment. In the WHO arbovirus definition [1,7], vertebrates play two roles in transmission, amplifying host and reservoir.

### 2.1. Historical Perspective

After the discovery of mosquito-borne transmission of the malaria parasite by Ronald Ross at the end of the 19th century, mosquitoes were called “carrier”, and humans were called “reservoir” for the agent of yellow fever (YF) [8]. The idea of identifying the source of the putative etiologic agent in an animal reservoir was largely established in the 1920s [9], with reports of animal hosts for Malta fever (brucellosis), plague, and human trypanosomiasis. These stimulated researchers of the YF Commission of the Rockefeller Foundation (RF) in West Africa and South America to look for the reservoir(s) of the etiologic agent [10].

The contributions of Karl F. Meyer and his interactions with prominent scientists at the time hold a position of pivotal importance in the birth of the transmission concept. Meyer, a strong advocate of the zoonosis concept of Rudolph Virchow, emphasized an intimate relationship between human medicine and veterinary medicine (now known by “One Health”). Through his communications with Theobald Smith, Macfarlane Burnet, Charles Elton, and George Nuttall, Meyer developed an interest in the vector-borne transmission of pathogens, latent infection, the equilibrium between virulence (or host immunity) and pathogen maintenance in nature, and ecological ramification in pathogen transmission [11]. He subsequently conceived an idea that the Animal Kingdom is the reservoir of microbial disease agents [12].

**Figure 1:**
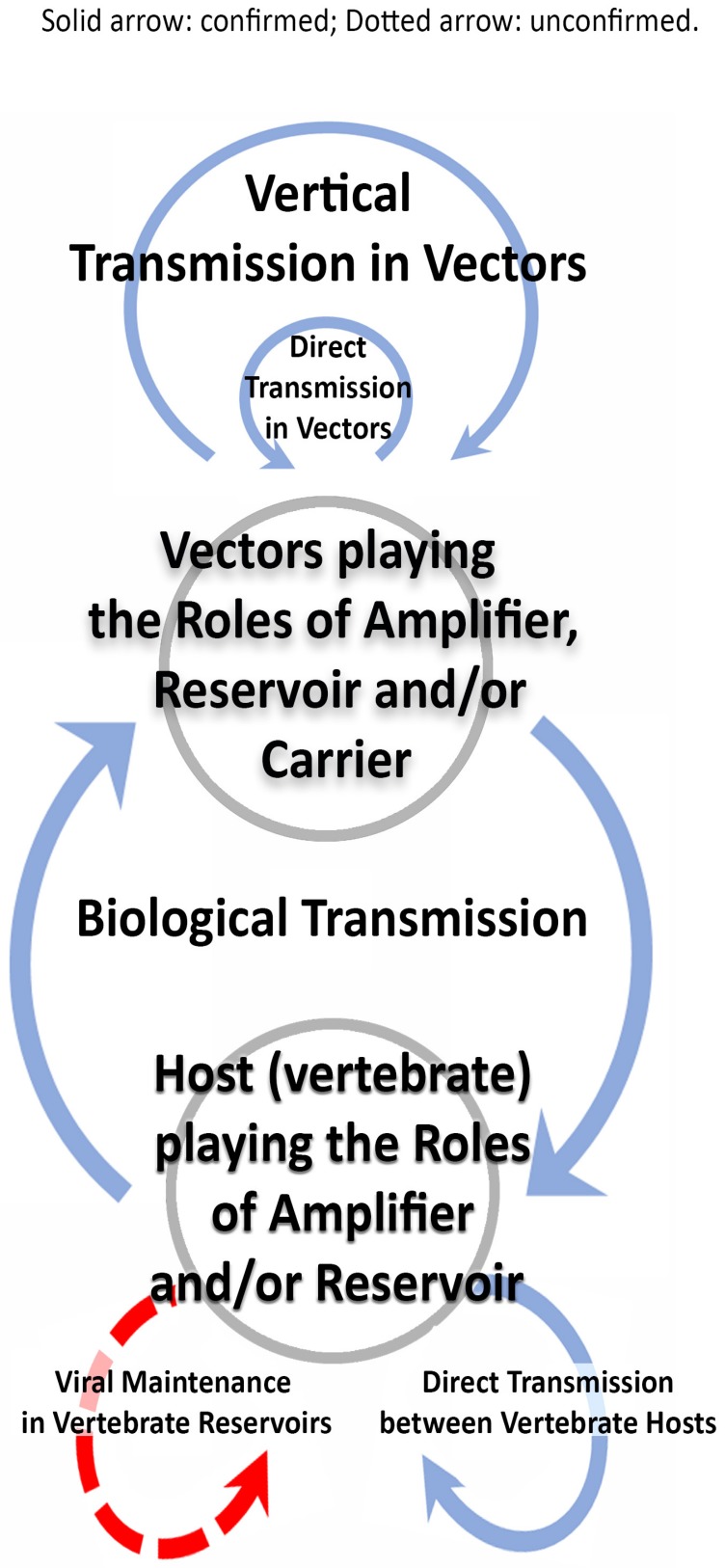
Generalized composite transmission cycle of RNA arboviruses.

To characterize vector-borne transmission of viruses, Meyer used “insect-borne disease” for YF, dengue, sandfly fever, and Rift Valley fever (RVF); “heterogeneous infection chain”, depicted in “cyclic organization” (currently “cycle”), to characterize the chain of transmission between arthropods and vertebrates; and “blind end” [13] (later “dead-end host” [14]) for the infected vertebrates which could not serve in further transmission. The arboviral replication which takes place in arthropods was termed “cyclopropagative multiplication,” with the arthropod playing the role in transmission as “carrier” or “vector” [12]. Among some investigators, an unpublished set of requirements was privately used to characterize the vertebrate reservoirs [15,16]. The term “biological transmission” (BT), which was originally coined to characterize the mode of transmission of parasitic pathogens [17], was adopted first for plant viruses [18] and later for arboviruses [19]. From these events evolved a set of conditions necessary to maintain the natural cycle of arbovirus transmission [10]. The major components of the transmission cycle are shown in Figure 1.

As for the identities of the reservoirs, initially multiple intriguing observations strengthened the notion of vertebrate reservoirs among early workers. First, a suspicion of the involvement of wildlife in the occurrence of YF was raised by Harold W. Thomas, who experimentally demonstrated yellow fever virus (YFV) transmission to a chimpanzee through the bite of mosquitoes after an extrinsic incubation period of 3 weeks [20]. The die-off of howler monkeys in Trinidad coincident with an outbreak of YF [21], the isolation of YFV in 1927 from monkeys by the RF researchers, the discoveries of jungle YF by Roberto Franco in Colombia and Fred L. Soper in Brazil between 1907 and 1932, arbovirus isolation from vertebrates, and detection of virus-specific antibodies in the suspected hosts on multiple continents were further indications supporting the notion.

Later, under laboratory conditions, Western equine encephalitis virus (WEEV) was found to persist for 234 days in a house sparrow [22], and bluetongue virus (BTV) persisted for as long as 1595 days in cattle [23]. If the length of persistent infection required to serve as reservoir was arbitrarily set at any length over one month, the number of laboratory results meeting this criterion would be large [24]. By the early 1960s, long term persistence of BTV in indigenous dwarf sheep and African horse sickness virus (AHSV) in zebras, both without much pathology, were speculated to be examples of arbovirus persistence [25]. A Russian report of West Nile virus (WNV) viremia for 100 days in a blue-gray pigeon was also suggestive [26].

### 2.2. Early Definition of Vertebrate Reservoir

The early definition of reservoir referred to an animal species (vertebrate) presenting a chronic or persistent infection with or without a pathology or loss of function (including reproductive potential) but shedding (into the blood, urine, feces, and/or bodily fluids) a sufficient amount of infectious agent to serve as a source of infection to members of the same host and to animals of other species (including humans and arthropods) [27,28,29]. Although the length of virus shedding could be short or intermittent [30], it was more often thought to last for the rest of the animal’s life. Later, latent infections, which normally do not shed a virus but which lead to shedding by activation, were also included [31].

### 2.3. Additional Evidence Necessary for Confirming a Vertebrate Reservoir for an Arbovirus and Data Incompatible with the Early Definition

#### 2.3.1. Additional Evidence

Because of the unique mode of transmission, definitive confirmation of an arbovirus’ vertebrate reservoir requires satisfying the following empirically-learned conditions between vectors and vertebrates [10,14,32,33,34,35] in a given location. These include: (i) either continual or intermittent isolation of an arbovirus in question from the suspected vertebrates during the period of complete cessation of vector’s feeding activity (a minimum of 2 but preferably ≥3 months) (hereafter called the “interrupted period”), such as during the dry season in the tropics or the cold season in temperate regions; (ii) the total absence of the introduction of infected vertebrates and/or vectors during the interrupted period; and (iii) the detection of the infectious virus from blood after the interrupted period. Ideally, a consensus among multiple groups of independent researchers for a given virus-host relationship is desirable. Furthermore, for a definitive confirmation, it is important to be able to distinguish if viral persistence represents a rare or frequent event under natural conditions.

In the following discussion, the early definition (Section 2.2) tailored to meet the additional evidence unique to arboviruses noted above is hereafter considered the “classical definition” of a vertebrate reservoir.

#### 2.3.2. Data Incompatible with the Early Definition

According to the classical definition, persistence of a disease or continual virus isolation in a given location (i.e., “enzootic” area) over years confirms persistent transmission or existence of the virus. However, it does not necessarily confirm existence of vertebrate reservoirs. Seroprevalence in suspected vertebrate reservoirs alone is not proof of reservoir status, though it confirms contact between the host and virus. For example, antibodies to insect-specific viruses have been found in serologic surveys of humans and other mammals, even though these viruses are not known to replicate in vertebrates [36,37,38]. Similarly, detection of virus-specific genome sequences in hosts by metagenomic techniques alone is insufficient to identify the hosts as reservoirs, as demonstrated by Ndumu virus (an alphavirus) or Gouléako virus (a bunyavirus) [39,40]. Virus isolation and replication studies are still necessary to determine the full host range and mode of transmission [41,42]. Other problematic issues are described in Section 3.7.

## 3. Problems

### 3.1. Unresolved Issues

Unlike for bacterial, protozoan, and helminthic pathogens, finding vertebrate reservoirs for arboviruses in nature becomes an elusive goal, because prolonged viremia in the vertebrate hosts is most often prevented by either vigorous immune responses (and hence shortening of the viremia period) and/or high mortality, characteristics unfavorable for vertebrate reservoir status by the classical definition. In fact, change or variation in the virulence of microorganisms in relation to the establishment of the latent infection or maintenance in reservoirs was the subject Meyer grappled with, but without a clear solution, according to one historian’s account [11]. In the following analyses of the troubling reports, it is important to consider the variation among vector–host relationships derived from differences in the origin of the arboviruses in question as one of the possible underlying causes.

In the most comprehensive study of vertebrates associated with arbovirus transmission in Africa and South America, the RF group could not find a vertebrate reservoir for YFV [10,14]. Similarly, in North America, vertebrate reservoirs for WEEV and Saint Louis encephalitis (SLEV) viruses could not be found [43]. In Asia, water birds were suspected to be the reservoirs of Japanese encephalitis virus (JEV), but subsequent investigations could not confirm this role for any avian host [44]. In South America, intense investigations revealed that small numbers of YFV vector species were still active during the dry season when vector activity was thought to cease [10].

Other sporadic reports of arbovirus isolations from wildlife during the winter could not be corroborated independently or the data were considered questionable because of the possibility of vector activity during unfavorable periods [33,34,45,46]. The question of vertebrate reservoirs for Rift Valley fever virus (RVFV) in Africa was solved by the discovery that the virus was maintained in the eggs of a flood-water mosquito vector during the dry period which may extend several years or more, depending on the weather pattern and/or location. In Australia, evidence indicated that Ross River virus (RRV) and Murray Valley encephalitis virus (MVEV) were able to persist in mosquitoes during the dry season in desiccation-resistant mosquito eggs [47,48]. California serogroup bunyaviruses are also known to overwinter in mosquito eggs in North America. In parts of South Africa, where it frosts for at least several weeks in mid-year, *Culicoides* midges (the vectors of AHSV) were continuously collected despite low densities [49]. Furthermore, in parts of the temperate regions virus-infected female mosquitoes (i.e., *Culex* spp.) are known to overwinter in a state of reproductive diapause [50].

Bats have been suspected as possible reservoirs of arboviruses since the early 1930s [51]. Many arboviruses have subsequently been isolated from a number of species, and laboratory experiments were conducted [52,53,54,55]. Despite this and many experimental laboratory infections, no bat has met the required reservoir criteria. Similarly, reptiles and amphibians have not been proven to be reservoirs, although alphaviruses were infrequently isolated from them in the past [56,57]. Numerous reports of arbovirus persistence under experimental conditions were found to be either not reproducible, irrelevant (because of the use of unnatural vertebrates or immunodeficient animals), or rare events, because no example meeting the requirements of viral persistence was ever found in the field [58,59,60,61,62].

### 3.2. Concerns Over the Validity

Richard M. Taylor concluded that mosquitoes qualify better as reservoirs than do vertebrates [14]. Critics either flatly rejected the notion because of the assumptions used or held serious reservations, recognizing deficiencies in the notion, demanding proof, or advocating alternative approaches [32,63,64,65,66,67]. It is important to note that when the zoonosis concept of the Joint Committee of the Food and Agriculture Organization (FAO) of the United Nations [68] and the arbovirus definition of the WHO [7] were simultaneously published in 1967, the FAO report was severely criticized for lack of ecological data and ambiguity about reservoirs for many pathogens considered zoonotic agents [69]. The shared problem in the WHO document was not surprising because the original advocate, Karl F. Meyer, who submitted the zoonosis concept to the General Assembly of WHO in 1954 [70], was also the principal architect of the arbovirus definition.

### 3.3. Conceptual Weakness

Two other facts have also contributed to the weakness of the notion. First, all RNA arboviruses cause acute infections in vertebrates; second, among other RNA viruses (including retroviruses) which chronically infect vertebrates, none replicates in or is transmitted by an arthropod vector in nature. Also, the characterization of chronic infections by others [71] excludes arbovirus infections. In another study, it was concluded that vector-borne viruses are not involved in inter-host maintenance because they cause higher mortalities, typical of acute infection, and do not establish a chronic infection [72]. Lastly, persistently-infected vertebrates have not been used even in simulated BT experiments, as described in Section 3.6.

### 3.4. Immunity

As described earlier (Section 3.1), because viral infection induces immune responses in vertebrate hosts, resulting in increased herd immunity, involvement of vertebrates of multiple species or those with high population turnover (in particular, small rodents and some birds) was suggested in the WHO definition to offset the loss of susceptible animals [7]. Because this suggestion contradicts the required trait (prolonged infection) for vertebrate reservoirs, it is best to interpret this as another mechanism of arbovirus maintenance by BT rather than as a reference to a shared trait of reservoirs.

Although immune responses lead to viremia clearance in most surviving hosts, infectious viruses (in particular, neurotropic viruses) may persist for longer periods (>2 years) in the brain, spleen, kidney and/or other organs either in the absence of neutralizing antibodies or when being shielded from exposure to them in the blood [73]. Also, it was reported that tick-borne encephalitis virus (TBEV) is transmitted from infective ticks (nymphs) to non-infected nymphs cofeeding on the same rodents with circulating neutralizing antibodies to the virus [74]. It should be noted that this phenomenon is observed only when infective and non-infected nymphs co-feed very close to each other on the same host [75].

It has been speculated that when the humoral immune response of the host is weak or if the virus in the immune complex is not completely neutralized, mosquitoes biting these vertebrate hosts may be infected, even with low frequency [76]. This possibility is compatible with the observation that cattle naturally infected with Middle Point orbivirus had prolonged viremia (up to 15 weeks) most frequently in hosts with low antibody levels [77]. Interestingly, Kyasanur Forest disease virus was re-isolated from the brains and liver of 14 of 18 mice demonstrating no neutralizing antibodies as long as 246 days after intracerebral or intraperitoneal inoculation [78]. This neutralization-based hypothesis [76] had been conceived earlier when Colorado tick fever virus was found to replicate for prolonged periods (up to 50 days) sequestered in the erythrocytes (and possibly in marrow) in hosts with adequate levels of antibody but without evidence of viremia [79,80]. Under experimental laboratory conditions, WNV immune complexes with anti-flavivirus antibodies remained infectious in vitro, albeit at a greatly reduced level [81]. It has also been reported that, though infrequently, dengue virus serotypes (DENV1, DENV2, or DENV4) could be isolated from patients who experienced a repeated infection by one serotype despite the circulation of neutralizing antibodies to it after the first episode [82,83]. These reports, however, must be interpreted with caution, because the putative first infection was determined by a neutralization test (using 50% instead of 90% plaque reduction criterion) that frequently detects heterotypic antibodies and not by virus isolation.

Emergence of neutralization-escape mutants was proposed as a possible mechanism of virus persistence. It was demonstrated through repeated exposure of the virus to a neutralizing monoclonal antibody and mutant selection under laboratory conditions [84,85,86]. Furthermore, a considerable variation in the magnitude of neutralization has also been reported among the virus isolates (temporally or geographically different) of arboviruses (such as DENV2, DENV3, louping ill virus (LIV), WNV, and WEEV) [87,88,89,90]. However, a definitive example of escape mutants contributing to arbovirus persistence has not been confirmed in natural systems where polyclonal antibodies react with the virus. Similarly, the significance of the immunosuppressing (and hence infection prolonging) role of the lymphoid cells (including specific T cells) in infected hosts in the context of vertebrate reservoirs remains unknown, even though demonstrable under laboratory conditions or in patients [91,92,93].

### 3.5. Persistence of Disease Symptoms

It is of great interest whether or not persistence of a particular disease symptom also suggests the persistence of the infectious virus. In the case of chronic polyarthralgia, infectious Chikungunya virus (CHIKV) persistence could not be established [94]. Polyarthralgia by RRV may last 3–6 months, but virus isolation from synovial fluid is possible only in the first 35 days after the onset of illness, despite persistent detection of viral RNA [95,96]. Though persistence or recurrence of symptoms has been reported more frequently in neurotropic arbovirus infections in animals, including WNV-infected parrots demonstrating neurologic symptoms for up to 6 years [97], infectious virus persistence has not been confirmed for non-neurotropic viruses. Explanations for the persistent symptoms include: (i) inflammatory responses to stimulation of the residual infectious virus due to incomplete clearance; and (ii) neurologic sequelae of progressively worsening inflammatory processes over a long period in the absence of the infectious virus [97].

In contrast, attempts to isolate TBEV from the brain of the victims of chronic TBEV infection occasionally yielded the infectious virus up to more than ten years after the onset of symptoms [73]. Similarly, JEV virus was isolated from the cerebrospinal fluid of a small number of patients more than 100 days after the onset of illness [98].

With the example of measles virus-induced systemic lupus erythematosus, explained by viral sequences being integrated into the patients’ genome [99], a question was raised if similar genome integration occurs in the patients of alphavirus polyarthralgia [100]. Since similar integration of fragments of arboviral RNA virus sequences in hosts is known [101], this possibility deserves investigation.

### 3.6. Laboratory Simulation

Because of the difficulties experienced in unlocking the complex virus transmission mechanism in nature, the WHO accepted partial reproduction of BT under laboratory conditions, although it was emphasized that final proof should be found in the field [1]. However, no one has ever reported cycles of transmission involving infected vertebrate hosts serving as reservoirs of arboviruses, even in a simplified laboratory setting.

### 3.7. Persistent Viral RNA vs. Persistence of Infectious Virus

More recently, persistence of viral RNA in experimentally-infected or naturally-infected vertebrates (including humans) has been reported in numerous publications. As examples, RNA of BTV was detected in the blood of goats for 2 years [102] and that of WNV was shed in the urine of a human for 2452 days [103]. Similarly, WNV RNA was found in the spleens of experimentally infected house sparrows for up to 36 weeks post infection [104] and in the brains of parrots for up to 6 years after the onset of symptoms [97]. In a study of overwintering of JEV in a temperate region of Asia, viral RNA was detected in one to two pigs per monthly sampling in the splenic mononuclear cells from January through March [105]. In another report, the persistence of TBEV RNA in placenta and embryos of voles 240–280 days after infection was interpreted as possible vertical transmission (VT) [106].

In all these field reports, the infectious virus was never isolated in the spring from the animals that had shed viral RNA for long periods during the winter months. As for the aforementioned patient who had detectable WNV RNA over six years [103], it should be noted that such long persistence of WNV RNA could not be corroborated in another study, despite a larger number of patients examined [107]. Furthermore, despite numerous reports of persistent viral RNA (but without an extended viremia), the type of RNA (full but non-infectious viral genome, viral RNA fragments, truncated defective RNA [108], RNA transcripts of host genome-integrated viral sequences [109], or something else) has not been determined, although a recent report suggests the possibility of the full length virus genome [97], which is supported by other reports of a quantitative increase of detectable viral RNA over the course of infection. As for the correlation between the persistence of WNV RNA in tissues and the presence of the infectious virus, in the studies of naturally-infected or experimentally-infected house sparrows the virus was not isolated from any of the birds [104], or it was isolated from the spleen 12 weeks after infection in only one of 34 RNA-positive birds. Collectively, the data in these and other similar reports reveal a lack of correlation and low probability of virus isolation during persistence of viral RNA exceeding 7 weeks [110].

Zika virus (ZIKV) is the only arbovirus confirmed to be sexually transmitted. The discovery of the viral RNA in semen for as long as 6 months after infection [111], coupled with reports of viral RNA persistence in placenta and congenital infection [112,113], raised the possibility of primates serving as reservoirs of this virus in nature [114]. Clearly, the answer depends on the definition of “reservoir”, and further studies are necessary.

## 4. Direct Transmission 

The number of reports of the occurrence of direct transmission (DT) by arboviruses is considerable [115]. Unusual modes of DT, such as infection of grouse by ingesting LIV-infected ticks [116] and fecal contamination with TBEV among ticks engaged in aggregated feeding [117] have been reported. Although DT is depicted in Figure 1, little is known if and how significantly it contributes to virus persistence.

Persistence of vesicular stomatitis virus serotype New Jersey (VSNJV) or serotype Indiana (VSIV) by DT was observed multiple times even when snow was on the ground [118]. Robert P. Hanson suggested temporarily-overlapping episodes of DT (including aerosol and oropharyngeal routes of infection) among animals confined to a limited space as the mechanism of the persistent occurrence of this virus [119]. Buggy Creek virus (BCRV) persists in the skin of house sparrows and is transmitted between birds under the congested condition of the nest [120]. Persistent detection of WNV RNA in birds during winter in the absence of mosquito activity [121,122] is also compatible with Hanson’s definition based on a temporarily overlapping chain of DT events by cannibalism. A recent report of JEV RNA persistence in oronasal secretions and in the tonsils of pigs for 25 days, despite a viremia that lasted less than 6 days [123], also raised a possibility of virus persistence by DT under highly congested conditions in swine breeding, even though the length of persistence was relatively brief. In West Africa and parts of Europe where there is no evidence of the involvement of ticks in the transmission of African swine fever virus (ASFV), not only feeding pigs virus-contaminated food (swill) but an unidentified DT mechanism between pigs is strongly suspected as being responsible for virus spread and/or maintenance [124,125].

## 5. The Problem of Assumption-Driven Research on Vertebrate Reservoirs and Its Consequences

Collectively, data from this review suggest that an assumption-driven approach has been applied for identifying the role of vertebrates as reservoirs when there was no supporting evidence. With the increasing agreement of the zoonotic concept of human diseases at the time, it is likely that the notion appeared highly plausible to many workers, and there has been an expectation that such reservoirs would eventually be discovered in the future. Unfortunately, there has been a shift of emphasis from field to laboratory-oriented research in recent years, driven partly by reduced financial support for field research and partly by the shift of interest among researchers [119,126,127,128].

During this process, the distinction between reservoir and amplifier has become blurred or the two terms have become synonymous [129,130,131]. Vertebrate hosts were functionally differentiated based on a combination of their habitat and the type of arboviral transmission, with reservoirs being involved almost exclusively in enzootic transmission in sylvan/rural environments and amplifiers in epidemic/epizootic transmission in urban/suburban environments [132].

Analyzing the consequences of these developments is even more complex because of a combination of paradox, puzzle, and irony—the paradox is that despite these new trends, the notion originally presented as a strong belief or hypothesis has become a quasi-integrated component of the transmission concept and has become deeply rooted worldwide; the major puzzle is that the aforementioned negative reports and serious concerns regarding vertebrate reservoirs expressed by leading scientists were not reflected in the WHO documents [1,7]; and the irony is that the validity of the notion has remained unresolved, and Meyer’s zoonotic concept was reborn in the form of “One Health,” which has become a respected and indispensable branch of infectious disease communication/research today [133].

## 6. Other Sources that Complicate an Understanding of the Notion

The ambiguity of the notion in the WHO concept spawned a variation of interpretations and inevitably a semantic confusion. Thus, the term “maintenance”, originally used to characterize the role of the virus reservoir acting as a population has been interpreted by some to mean a chain of BT ensuring the perpetual infection of hosts, presumably in an environment without a temporal interruption of vector activity.

Nearly all authors have commented on transmission mechanisms with a clear functional difference in mind between amplifying host and reservoir. However, difficulties in deciphering which of the two roles the animal in question plays may be complicated by a combination of: (a) the absence of true reservoirs that meet the classical definition; (b) variation in the required criteria for vertebrate reservoirs; and (c) the meaning of “maintenance” (such as perpetuation of BT, maintenance of virus in the reservoir, or the survival of the virus by a combination of all mechanisms) [134].

More recently, as the term “reservoir” began to be used only for the hosts primarily involved in BT, their capacity to transmit was measured by an index analogous to “vector competence” for vectors. Unfortunately, these indices proliferated without a definition of “reservoir”, and yardsticks such as “reservoir competence,” “reservoir index,” or other indices determined on the bases of proportion of susceptible hosts, infectiousness to vectors, mean duration of viremia, seropositivity, reproduction number, and/or blood feeding and transmission activities of vectors [131,135,136,137] related more to an “amplifier index” than to the reservoir host as defined by the early or classical definition. In the meanwhile, the early definition was also interpreted by some to mean that reservoir hosts could be re-infected several times during their lives [132].

Another possible factor that could have contributed to the confusion about the notion of vertebrate reservoirs relates to the breadth of content of the WHO document which covered both arboviruses and rodent viruses (hantaviruses and arenaviruses) [1]. Because of this, information about small vertebrates as reservoirs of arboviruses is followed by a paragraph emphasizing rodents as reservoirs of rodent viruses. Thus, it is possible that to some workers the WHO’s notion of arboviruses did not appear unusual or questionable.

## 7. Origins of Arboviruses

The notion of vertebrate reservoirs is an essential component in understanding the origins of arboviruses. Traditionally, it was thought that arboviruses derived from vertebrate viruses, as exemplified by a discussion of zoonoses [138,139] and in the title of an authoritative book [140]. However, in the early studies of arthropod-borne viruses, the idea of an arthropod origin was actually more common [34,141,142,143,144,145].

William C. Reeves and his colleagues were probably among early proponents of the arthropod origin of arboviruses, stating that California encephalitis virus (CEV) “is probably a mosquito virus that is partially adapted to a narrow range of vertebrate hosts but is not reliant on vertebrates for its continuous existence” [146]. This mode of arbovirus maintenance roughly resembles the “basic maintenance cycle” of arboviruses which is maintained without a heavy dependence on epidemic or epizootic transmission by BT, according to Roy W. Chamberlain [66]. Some investigators, on the other hand, speculated that arboviruses might have a number of origins in vectors and/or in vertebrates [147].

### 7.1. Recent Developments

A large number of arthropod viruses belonging to multiple virus families and sharing their genome organization with the corresponding arbovirus members in each genus or family have been described in recent years, necessitating a modification of the traditional (WHO) arbovirus definition [148,149,150,151]. Other relevant discoveries include integration of the segments of the arbovirus genome into the vector genome and isolations of insect-specific viruses (non-arboviruses) sharing parts of genome sequences and other traits with the viruses in the families outside the five traditional arbovirus families, essentially presenting genomic mosaics. This new information provides further evidence of possible extensive involvement of arthropods in the evolution of arboviruses and other animal viruses [152,153,154,155,156,157,158,159]. In addition, phylogenetic studies show the positions of arthropod-specific viruses to be at or near the roots of genus or family trees. Furthermore, each branch in a given tree corresponds to a specific group of arthropods rather than to a vertebrate group [148,160,161,162,163,164,165,166].

The importance of analyzing trees based on empirical markers has long been recognized to infer the origins of animal viruses, given the lack of traditional fossil evidence [167]. In phylogeographic or epidemiologic analyses, geologic events (including tectonic shift) and the birth of settled human population centers associated with the rise of agriculture have been commonly used as substitutes for empirical markers; but in a study that determined the origin of the family *Bunyaviridae* in arthropods, properties of virions (including glycoproteins and RNA polymerase) and host range were found to be useful phenotypes [42]. A similar conclusion was obtained by others independently [168,169].

Since arboviruses represent heterogeneous animal virus families, it is also possible that some lineages could have derived from organisms other than arthropods. A survey revealed that vertebrate viruses not known to be arboviruses but which replicate in invertebrates represent many virus families [170]; and a recent study revealed a complexity of virus–host relationships, and hence a history of host range shift among RNA animal virus families [151].

Even among arboviruses, several alphaviruses have been known to replicate in the cells derived from fishes; and conversely, some aquatic viruses (including some fish viruses) replicate in the cells derived from terrestrial arthropods [170]. Thus, it has been speculated that the ancestor of alphaviruses may be a fish virus [171]. To further complicate the study of the origins of arboviruses, Flock House virus (a nodavirus, family *Nodaviridae* and not an arbovirus) replicates in insects (including mosquitoes), vertebrates, yeasts, nematodes, and plants. Members of the family *Birnaviridae* infect vertebrates, arthropods, mollusks, and/or nematodes; and members of the largely fungal and protozoan virus family *Totiviridae* infect mosquitoes and salmonid fishes; some of them can infect mammalian cells as well [170].

Finally, some important questions remain unanswered for one of the most comprehensively studied virus groups: the genus *Flavivirus*. First, does Tamana bat virus (TABV) belong to this genus or to a new genus yet to be created? Second, what are the taxonomic positions of flavivirus-like viruses isolated from non-hematophagous arthropods (i.e., segmented jingmenviruses) [159], a nematode, a plant, and aphids? Third, at which node in the inferred phylogenetic tree did “arbovirus members” originate? The answers to these questions depend largely on the viruses used as outgroup and the selection of the viruses to be included in the tree. This is especially important for determining branching order for virus lineages revealing repeated and drastic host range shifts in their histories.

### 7.2. Vectors as Reservoirs

The fact that ticks play the role of reservoir in the transmission of tick-borne viruses was recognized early in the 1930s and later incorporated in the arbovirus definition of the WHO. However, recognition of mosquitoes as reservoirs was considerably delayed. As an example, natural VT of YFV by *Aedes aegypti* mosquitoes was reported in 1903, but the first confirmation was reported only in 1997 [172], after many failures to reproduce the results. That the main reservoir of YFV in South America was *Haemagogus* mosquitoes in sylvan environments [70] was also confirmed much later [173]. However, by early 1960s the idea of vectors as reservoirs gained ground [174]. In Russia, WNV is known to overwinter not only in mosquitoes but also in ticks [175]. Currently, VT has been confirmed for a large number of arboviruses in the field and under laboratory conditions [176].

RNA arboviruses replicate in vectors generally without a noticeable pathology at a population level, although pathologies and reduced fecundity were reported for several viruses at the individual level. In addition to VT (transovarial and transovum modes of transmission), DT (such as venereal transmission, intergenerational virus transfer between co-feeding ticks, and oral infection of mosquito larvae) is known to occur. Furthermore, the importance of overwintering of infected female mosquitoes [50] needs to be recognized. Langat virus (LGTV) may persist in ticks for at least 8 years under laboratory conditions and ASFV in individual adult ticks for at least 2.4 years, while BCRV persists in cimicid bugs for 2 years [177,178,179].

Recent reports on the mechanism(s) facilitating persistent arbovirus infection without significant pathology in vectors have raised the possibility that DNA forms of DENV2, WNV, La Crosse virus (LACV; an orthobunyavirus member of California encephalitis virus serogroup), and CHIKV integrated as fragments in the vector genome facilitate persistence [180,181]. This has been established as one of the mechanisms of latent infection of other vertebrate viruses which facilitate viral replication without significant pathologies to the host cells.

The information presented in this subsection should not be interpreted to suggest that, like arthropod-specific viruses, arboviruses are maintained in infected populations of vectors alone since the maintenance mechanism involving multiple interacting parameters is complicated and, by definition, true arboviruses require vertebrates.

## 8. Arguments for and against the Existence of Vertebrate Reservoirs

- The definition of reservoir itself has been a source of disagreement. First, whenever an original definition is found to be unrealistic, a pragmatic definition compatible with reality must be adopted. Furthermore, because arboviruses are a heterogenous group of viruses, it would not be unexpected if the mechanisms of viral maintenance in nature are also heterogenous. Accordingly, the application of a single reservoir definition for all arboviruses is inappropriate.
- Because multiple vectors and/or multiple hosts are involved in the transmission of most arboviruses, identification of vertebrate reservoirs is complicated [33,57]. For example, because of enormous complexity the identification of the natural maintenance mechanism of YFV, probably the most intensively studied arbovirus, the mechanism has remained unknown even in an island of limited land mass (Trinidad) [182]. Also, enzootic foci of most viruses, with some exceptions characterized by well-defined focal distribution, do not remain indefinitely at any fixed location within a vast enzootic zone, rendering establishment of field sites difficult. In reality, most attempts to search for reservoirs have been incomplete in one ecological respect or another. This is a shared problem in all branches of zoonotic and parasitic disease research [183]. Even for ebolaviruses, which are vertebrate viruses, the definitive identities of the reservoirs have remained elusive despite arduous research for many years. However, bats are now suspected because of the detection of viral RNA and high rates of seropositivity [184,185]. Furthermore, the impossibility of colonizing some local vectors and/or suspected wildlife has prevented the completion of laboratory experiments.
- The recent discoveries of new viruses with unexpected viral traits and the host range necessitate redefining many of the basics of arbovirology. Accordingly, it is too early to preclude the existence of vertebrate reservoirs, because such examples might be discovered in the future. In fact, Mokola virus, a member of the *Lyssavirus* genus (family *Rhabdoviridae*), is known to replicate in mosquitoes and produces viremia in mice, although the vector-borne mode of transmission for this virus has never been established [186]; nor has its vertebrate reservoir(s) been identified. Also, three bat-associated flaviviruses (Entebbe bat virus, Sokuluk virus, and Yokose virus), which are not vector-borne, replicate in mosquito cells [187,188].
- Identifying vertebrate reservoirs is a difficult task, because persistently infected hosts are presumed to demonstrate little or no sign of illness according to the notion. Viral latency is sometimes confirmed only by applying an immunosuppressant to activate the virus or by blindly co-cultivating tissue samples obtained from asymptomatic animals in a highly sensitive cell culture. But, for arboviruses, such a laborious blind test has rarely been applied routinely to asymptomatic wildlife, except in a small number of experimental studies [24]. Furthermore, the significance of persistent viral RNA without the isolation of the infectious virus in terms of reservoir status is still poorly understood. Thus, vertebrate reservoirs remain yet to be discovered.

## 9. Current Knowledge of the Roles of Vertebrates and Vectors

- The role of DT by vertebrates in the maintenance of arboviruses is poorly understood.
- No vertebrate host of RNA arboviruses satisfies the required qualities of reservoirs based on the classical definition.
- The only arbovirus whose vertebrate hosts satisfy the classic definition is tick-borne ASFV, a DNA arbovirus [189,190].
- Vertebrates serve as a source of blood for vectors and as an amplifier for arboviruses, whether the environment in question be sylvan/rural (as in enzootic cycle) or urban/suburban.
- For those arboviruses for which the vector-vertebrate host relationship is well established, vectors serve as reservoir, amplifier, and carrier, whether the environment in question be sylvan/rural or urban/suburban.
- The vertebrate host range of an arbovirus is determined principally by the vectors involved in transmission and the availability of the hosts in a particular ecosystem. Depending on the virus, the range may expand to secondary hosts and/or vectors, as a result of selection pressure on the virus generated either in vectors or in hosts as well as of the degree of promiscuity of host seeking behavior of the vectors involved.

## 10. Impact of Molecular Virologic Research

According to one of the prevalent theories regarding arbovirus origin, DENV1, DENV2, DENV3, DENV4 and YFV originated from subhuman primates, because virus transfer to humans was facilitated by the closer genetic distance between monkeys and humans; other zoonotic arboviruses evolved from an adaptive host range shift of enzootic vertebrate viruses to humans [29,191,192]. On the other hand, others have hypothesized that the dengue viruses originated in mosquitoes and jumped first to primates in the forest and then to humans [193,194]. This hypothesis is supported by the fact that both non-human primates and humans act as amplification hosts for these viruses, not reservoirs.

As in all other viral lineages demonstrating a history of considerable host range shift (such as lyssaviruses and hantaviruses [169,195]), identification of reservoirs is crucially important for determining the direction of host range change. Studying the history of host range shift among arboviruses similarly provides information as to how viruses of a narrow host range (specialists) such as DENV1, DENV2, DENV3, and DENV4 evolved among arboviruses mostly known for host range plasticity (and hence are generalists) [196]. Another important aspect of arboviruses in the context of host range and with respect to emerging infectious diseases is that while host range shifts (including spillover phenomenon) observed in most vertebrate viruses concern the shift between species within a genus or family of mammals, the adaptation of arboviruses in two phyla of organisms (vertebrates and invertebrates) represents a far greater magnitude of shift.

Thus, it would be worthwhile reexamining the existing theories or conclusions of past research to re-assess if or how the new sets of information described in Section 7 and Section 9 impact the existing theories or conclusions of past research. The selected research questions for this exercise are as follows:

(a) Phylogenetic studies to identify the origin of arboviruses and evolutionary direction in the context of the vector/host range shift.
(b) The direct correlation between virus genomic traits and the host range, such as codon usage bias and principal component analysis, bypassing the involvement of vectors [197,198,199].
(c) Molecular determinants of viruses involved in the vector or host range shift.
(d) If phylogenetic studies can be used to predict the establishment of the endemic urban transmission of CHIKV, ZIKV and YFV in the future.

Regarding the research question (c), molecular determinants identified in Venezuelan equine encephalitis virus (VEEV) and CHIKV thus far correlated with the vector shift [200,201], while other studies revealed the role of hosts in the emergence of adapted strains of WNV [202] or of quasi-species of vector mosquito for CHIKV [203]. BCRV is known to have two lineages, one relying more on BT and the other mostly on cimicid bugs (vectors) [204]. Thus, this virus provides an opportunity to compare adaptive mutations in vectors and hosts at the molecular level, because of the simple vector–host relation involving only one species of vector and two vertebrate species (swallows and house sparrows).

Research question (d) was raised from a conclusion that sylvan and urban strains of four serotypes of dengue virus fall into separate clades, indicating insignificant gene flow between clades [205,206]. This did not necessarily preclude a possibility of sylvan strains causing dengue in an urban setting [207]. As for YFV, sylvan and urban strains of this virus are mixed, indicating that the former is specific to the main reservoirs in sylvan environments and that lineages of this virus capable of establishing a perpetual urban endemicity have not evolved yet [206].

## 11. The Impact of Epidemiology/Epizootiology, Disease Prevention/Control, and Zoonoses

### 11.1. Epidemiology/Epizootiology

As in the previous section, a similar exercise would be useful to reassess the impact on the current epidemiologic/epizootic questions. Ideally, a number of research questions below can be asked in the holistic context of virus–vector–vertebrate interactions:

- Why YF does not occur in tropical Asia infested by the principal vector, *Ae. aegypti* [208], a century-old unresolved question and a topic of growing interest in the wake of importation of YF cases to China from Africa [209]. This in spite of the fact that Asian strains of *Ae. aegypti* mosquitoes are competent in the transmission of this virus [210,211] and some Asian monkeys (such as rhesus monkeys) are susceptible to infection by YFV.
- Why JEV has not spread to Africa and the Middle East where the principal vector, *Culex tritaeniorhynchus*, has been known to be distributed for a long time. A recent report of detection of JEV RNA in a patient in Angola [212] is of interest.
- Why has dengue endemic transmission not been established in temperate parts of Asia infested by a vector, *Ae. albopictus*, despite its superior vector competence measured under laboratory conditions [213]? This is explained possibly in part due to its lower dependence on human blood and its less efficient virus dissemination in the body of *Ae. albopictus* mosquito [214].
- If ASFV is indeed spreading in parts of Africa and Europe only by DT, what is the mechanism involved?
- If CHIKV and ZIKV will establish an enzootic cycle in sylvan/feral areas of the Americas, Australia, or Europe.
- Of the seven viruses (DENV1, DENV2, DENV3, DENV4, CHIKV, YFV, and ZIKV) transmitted by the same vector (*Ae. aegypti*) in an urban cycle, why is it that only the four serotypes of dengue virus can maintain perpetual urban transmission cycles and the other viruses disappear from a given urban center of considerable population size after less than 5 years of transmission? The answer to the latter question might provide an explanation for the presumed failure of CHIKV to establish endemicity in the 19th century in the Americas, if in fact the controversial retrospective speculation of a chikungunya pandemic in that century [215] is correct. In contrast, WNV readily established endemicity there after the 1999 invasion, most likely because of broad vector and vertebrate host ranges. A similar phenomenon was observed in the global spread of BTV and of Usutu virus spread in Europe.
- Are there two mechanisms used by arboviruses for maintenance, one based on BT and the other largely based on DT? This possibility was raised on the basis of long-term surveillance of VSNJV activity in an island where the number of visitors is limited and human activities are severely-controlled [216], an ideal environment for studying the previously mentioned “basic maintenance mechanism [66]”.

### 11.2. Disease Prevention/Control

The importance of vector control in mitigating arboviral diseases remains the same regardless of the difference in the definition of the vertebrate reservoir. However, other concerns about the notion relate to planning a strategy for preventing the introduction into or controlling the spread of an exotic arbovirus to a virgin territory or controlling its spread, such as the introduction of RVFV into North America [217]. As pointed out in the report, planning a control strategy is difficult because of its known broad vertebrate host range and not knowing on which potential animal reservoirs to focus, given the fact that this is unknown even in its native Africa.

Culling and restricting movement of domesticated animals have been two of the traditional methods for disease control in agriculture. The idea of culling wildlife populations to control arbovirus epizootics [218] is a more difficult proposition, as shown in a program to control LIV in Scotland where the objective of control is not to protect sheep but to protect grouse (wildlife), which is economically more important as a game bird [219].

### 11.3. Zoonoses

In light of the growing number of reports supporting the arthropod origin of many arboviruses, the adequacy of the traditional practice of applying the term “zoonosis” to human infections by these viruses is another subject for reappraisal. Vertebrate reservoirs are generally thought to be the donors of newly emerging viruses involved in spillover [139]. However, involvement of both vectors and vertebrate hosts complicates identification of the donors for arboviruses and demonstrates the pitfalls of classifying zoonoses (including arboviral diseases) based on the source of the pathogens [220]. Accordingly, some researchers have begun to use the term “metazoonosis” for all vector-borne pathogens whose maintenance cycles require both vertebrates and invertebrates [221].

## 12. Options for a Solution

The objective of traditional vertebrate reservoir research has always been to satisfy the classical definition. More recently, other methods derived from various interpretations of the notion or definition (mentioned in Section 5 and Section 6) have been used. This is not unique to arbovirology, since various definitions have evolved over time pertaining to pathogen–host relationships, and have often been controversial [222]. A definition of “reservoir” in virology is no exception, as illustrated by the variation among its branches, ranging from specific T cells of patients harboring human immunodeficiency virus or macrophages in CHIKV-infected monkeys [223] to the ocean in the context of virosphere.

### 12.1. Revision of the Notion and Redefining Vertebrate Reservoir

To resolve many of the issues identified in this review, dropping the notion of vertebrate reservoir altogether is one option. However, because the terms “amplifier” and “reservoir”, as well as the corresponding criteria defining them, are different, we recommend a modification of the term and revision of the WHO definition as a more practical solution. By either the early or classical definition, viruses in reservoirs persist by relying on mechanisms other than BT; while viruses in amplifiers heavily rely on BT.

Alternative definitions of vertebrate reservoirs of arboviruses include Hanson’s temporarily overlapping episodes of DT and the ecosystem supporting perpetual maintenance of the virus as a whole. Reservoirs have also been defined as “vertebrates that carry viruses passively” [224]. As it is difficult to verify viremia in nature, eliminating persistent viremia from the required criteria is yet another possibility. Replacing “reservoir host” with “maintenance host” without a strict adherence to the early definition is another alternative. Including mechanical transmission (which is excluded in the WHO’s arbovirus definition) and recurrent (or annual) reintroduction of viruses also deserves serious consideration for a broader definition. For arboviruses in urban transmission cycles, such as DENV1, DENV2, DENV3, DENV4, CHIKV, and ZIKV, a subpopulation of infected humans may be considered a “transient reservoir” because of their rapid mobility facilitated by modern transportation systems. The other question is whether one definition for all arboviruses is adequate, given the fact that they represent a diverse group of viruses each with a distinct history of host association. Other alternative definitions, as well as associated issues, have been discussed elsewhere [225]. Whatever definition is adopted for arboviruses, dynamic, qualitative, and quantitative changes must be integrated into the term, to account for the heterogeneity in arbovirus transmission in time and place.

### 12.2. Online Communication in the Context of One Health

We suggest the establishment of an online forum to continue dialogue on all issues surrounding arbovirology (including reservoir). Soliciting opinions globally for more efficiently pooling ideas and data on arboviruses would be helpful at a time when the number of investigators in the field and research support are declining. Such an online system could also serve as a vital source of consultation for the immediate outbreak response network of the WHO [226].

This forum should be managed by an international committee representing not only arbovirologists, but also scientists from allied branches of virology (such as veterinary, invertebrate, and plant virology), medical entomology, tropical medicine, infectious disease research, public health, livestock trade organization, wildlife conservation, and/or zoonotic disease research. This multidisciplinary approach is favored because of a growing interest in the concept of “One Health” and the increasing frequency of pandemics and panzootics which threaten the health and economic security of many countries. After a period of debate, the committee would be expected to submit recommendations to a designated international body, such as the WHO, the American Committee on Arthropod-Borne Viruses (ACAV), or the International Committee on Taxonomy of Viruses.

## 13. Concluding Remarks

The year 2016 marked the centennial of the commencement of the Rockefeller Foundation’s field studies led by William C. Gorgas to conduct surveillance of YF outbreaks, and to identify and destroy the “seed beds” (now “endemic or enzootic foci” including reservoirs). We have just entered the second century of vertebrate reservoir research without success in identifying such reservoirs, as judged by the original definition. Regrettably, due to a combination of factors including the troubling notion itself and the declining support for field investigations, RNA arboviruses have become, to use a colloquial expression, “viruses in search of a vertebrate reservoir.”

Consequently, it is not difficult today to find new publications in which subhuman primates are still identified as reservoirs of YFV. Recently, a reservoir role of bats for DENV1 has been suspected merely because antibodies to and RNA of the virus were detected [227]; moreover, neotropical primates are now suspected to be reservoirs of ZIKV in Brazil, because of the detection of the viral sequences by real time PCR [228]. Furthermore, among some workers, the role of vectors is still interpreted exclusively as carrier, but not as reservoir [229].

The recent increased interest in maintenance mechanisms, reservoirs, and persistent infection of arboviruses [230,231,232,233,234,235,236,237,238,239], as well as in the issues concerned with defining a “vector” [240], underscores the importance of these topics. Thus, this review provides timely information to aid further discussion.

It is stressed that in any branch of science the application of multiple definitions for a key term is a serious impediment to scientific communication, leading to unnecessary confusion. When the validity of the original (and hence the principal) definition itself is questionable, then the complexity of the problem is magnified. The late William C. Reeves, when confronted with an unexpected outbreak of St. Louis encephalitis, left an apt phrase: “the heart of research is the reexamination of accepted concepts on the basis of new facts” [241]. Clearly, the time to redefine the roles of vertebrate hosts is long overdue in this branch of virology.

## Figures and Tables

**Table 1:** A list of vector–host relations of the arboviruses and related viruses referenced.

Virus Family	Virus (Abbreviation)	Vector(s)	Major Vertebrate Host(s)
*Asfarviridae*	African swine fever virus (ASFV)	ticks	pig; warthog; bushpig
*Bunyaviridae*	California encephalitis virus (CEV)	mosquitoes	?
	La Crosse virus (LACV)	mosquitoes	rodents
	Rift Valley fever virus (RVFV)	mosquitoes	bovine; goat; sheep; ruminants
	Toscana virus (TOSV)	sandflies	sheep; human?
*Flaviviridae*	Bagaza virus (BAGV)	mosquitoes; midges	birds
	Dengue virus serotypes (DENV1, DENV2, DENV3, DENV4)	mosquitoes	primates (monkeys and humans)
	Entebbe bat virus (ENTV)	-	bats
	Japanese encephalitis virus (JEV)	mosquitoes	birds; pig; horse
	Kyasanur Forest disease virus (KFDV)	ticks	rodents; monkey
	Langat virus (LGTV)	ticks	rodents
	Louping ill virus (LIV)	ticks	grouse; sheep
	Murray Valley encephalitis virus (MVEV)	mosquitoes	ardeid water birds
	Sokuluk virus (SOKV)	-	bats
	St. Louis encephalitis virus (SLEV)	mosquitoes	birds
	Tamana bat virus (TABV)	-	bats
	Tick-borne encephalitis virus (TBEV)	ticks	rodents
	West Nile virus (WNV)	mosquitoes	birds; horse
	Yellow fever virus (YFV)	mosquitoes	primates
	Yokose virus (YOKV)	-	bats
	Zika virus (ZIKV)	mosquitoes	primates
*Reoviridae*	African horse sickness virus (AHSV)	midges	horse; donkey; mule
	Bluetongue virus (BTV)	midges	ruminants
	Colorado tick fever virus (CTFV)	ticks	rodents
	Middle Point orbivirus (MPOV)	mosquitoes?	cattle
*Rhabdoviridae*	Bovine ephemeral fever virus (BEFV)	midges; mosquitoes	bovine
	Vesicular stomatitis virus—New Jersey serotype (VSNJV)	sandflies; blackflies; midges	bovine; swine; equine
	Vesicular stomatitis virus—Indiana serotype (VSIV)	sandflies; blackflies; midges	cattle; swine; equine
*Togaviridae*	Buggy Creek virus (BCRV)	cimicid bugs	cliff swallow; house sparrow
	Chikungunya virus (CHIKV)	mosquitoes	primates
	Ndumu virus (NDUV)	mosquitoes	cattle
	Ross River virus (RRV)	mosquitoes	marsupials; horses
	Venezuelan equine encephalitis virus (VEEV)	mosquitoes	rodents
	Western equine encephalitis virus (WEEV)	mosquitoes	birds; equine
